# Identifying the Shared and Dissociable Neural Bases between Self-Worth and Moral Ambivalence

**DOI:** 10.3390/brainsci14070736

**Published:** 2024-07-22

**Authors:** Jiwen Li, Shuai Wang, Tengfei Du, Jianchao Tang, Juan Yang

**Affiliations:** 1 Faculty of Psychology, Southwest University, No. 2 Tiansheng Street, Beibei District, Chongqing 400715, China; lijw0022@email.swu.edu.cn (J.L.); wangshuai@email.swu.edu.cn (S.W.); dtf001121@email.swu.edu.cn (T.D.); t1099304172@email.swu.edu.cn (J.T.); 2 Key Laboratory of Cognition and Personality, Southwest University, Chongqing 400715, China

**Keywords:** self-worth ambivalence, moral ambivalence, resting-state fMRI, superior parietal lobule, orbitofrontal cortex, temporoparietal junction

## Abstract

Self-ambivalence, a prevalent phenomenon in daily life, has been increasingly substantiated by research. It refers to conflicting self-views and evaluations, primarily concerning self-worth and morality. Previous behavioral research has distinguished self-worth and moral ambivalence, but it remains unclear whether they have separable neural bases. The present study addressed this question by examining resting-state brain activity (i.e., the fractional amplitude of low-frequency fluctuations, fALFF) and connectivity (i.e., resting-state functional connectivity, RSFC) in 112 college students. The results found that self-worth ambivalence was positively related to the fALFF in the orbitofrontal cortex (OFC) and left superior parietal lobule (SPL). The RSFC strength between the SPL and precuneus/posterior cingulate cortex (PCC) was positively related to self-worth ambivalence. Moral ambivalence was positively associated with the fALFF in the left SPL (extending into the temporoparietal junction) and right SPL. The RSFC strengths between the left SPL/TPJ and OFC, as well as the RSFC strengths between the right SPL as a seed and the bilateral middle and inferior temporal gyrus, were associated with moral ambivalence. Overall, the neural bases of self-worth and moral ambivalence are associated with the SPL and OFC, involved in attentional alertness and value representation, respectively. Additionally, the neural basis of moral ambivalence is associated with the TPJ, responsible for mentalizing.

## 1. Introduction

Self-ambivalence refers to the state of having conflicting or contradictory feelings, thoughts, or attitudes toward oneself [1]. It involves experiencing mixed emotions or holding opposing beliefs and opinions about one’s own identity or abilities [2]. Previous research viewed self-ambivalence as an abnormal psychological phenomenon found in individuals with obsessive–compulsive disorder and schizophrenia [3,4]. Recently, a growing body of evidence has uncovered the prevalence of self-ambivalence in healthy populations [5,6,7]. Our research further discovered that one aspect of self-ambivalence could be beneficial to one’s mental health.

Early evidence found that people experience conflicting beliefs and feelings about different aspects of the self, such as self-worth, morality, and lovability [1,8]. Self-worth ambivalence reflects a state of internal conflict about personal value, which mainly comes from uncertain beliefs about one’s ability or talent (i.e., I constantly worry about whether I will make anything of my life). Moral ambivalence reflects a state of conflict over one’s moral identity (i.e., I question whether I am morally a good or bad person). It involves incompatible beliefs about one’s morality. Lovability reflects an individual’s concern about being accepted and approved by others (i.e., I am constantly aware of how others perceive me). Compared with self-worth and moral ambivalence, lovability emphasizes the ambivalent attitudes toward the social image of the self. Self-worth ambivalence represents a global sense of self-worth [2]. Morality and lovability involved two specific domains of self-ambivalence.

The Self-Ambivalence Measure (SAM) is a tool frequently used to assess the construct of self-ambivalence in clinical and non-clinical populations [1]. Bhar et al.’s study was the first to use the SAM and identified two dimensions of existence, self-worth and moral ambivalence. Subsequent research found a third factor in the larger healthy population, named public self-consciousness [2], which reflects concerns about public self-image. Our recent study also found a three-dimensional structure for the presence of a SAM in Chinese populations. It is worth noting that the impact of public self-consciousness on mental health can be disregarded compared to self-worth and moral ambivalence [9]. Self-worth and moral ambivalence remained central to the field of self-ambivalence research [10].

There is evidence to indicate that self-worth and moral ambivalence represent related but unique components of self-ambivalence [10]. On one hand, both describe the thoughts and feelings of inner conflict, which contradicts self-enhancement motivations [11], thereby potentially causing psychological problems such as obsessive–compulsive symptoms and anxiety [12,13]. On the other hand, self-worth and moral ambivalence emphasize the different facets of self-ambivalence. Self-worth ambivalence represents uncertainty about self-worth, which is derived from abilities and personality [14]. Moral ambivalence represents uncertainty about the moral self [8]. They play different roles in predicting various aspects of obsessive–compulsive disorder [10]. Our results found that higher self-worth ambivalence was related to lower life satisfaction and happiness, while higher moral ambivalence was related to higher life satisfaction and happiness [9]. What remains unclear is how the similarities and differences between self-worth and moral ambivalence are represented on a neural basis. Research on this issue can help people understand the underlying cognitive processing of self-worth and moral ambivalence.

Several studies from task-based functional magnetic resonance imaging (fMRI) have found that brain regions responsible for conflict monitoring and conflict resolution are recruited when people are making ambivalent decisions [15,16,17]. These brain regions include the dorsal part of the anterior cingulate cortex (dACC), which was identified as a key region in monitoring conflicting information, and the dorsal lateral prefrontal cortex (dLPFC) and orbital frontal cortex (OFC), which are responsible for conflict resolution [18]. Self-ambivalence emphasizes that people are perceived as either positive or negative at the same time, without a means of reaching consistent knowledge [1]. It is clear that, unlike decision-making tasks, self-ambivalence focuses on the conflicting views of the self, whether the conflict is resolved or not. Thus, the monitoring of self-incompatible information should be the underlying cognitive process of self-ambivalence. In addition, the cognitive process of self-ambivalence should also include self-evaluation, which is a prerequisite for generating conflicting beliefs. The cortical midline structures (CMS) are considered the core region for self-cognition and self-evaluation, which was confirmed by task and resting-state fMRI studies [19,20,21,22].

Based on the moral self theory [23], a distinctive characteristic of the moral self is its pronounced social attributes. In addition to core personal characteristics, social factors of interactions with others are antecedents to the formation of the moral self concept [24]. For example, praise for moral behavior would change an individual’s evaluation of the moral self. Research supports the assumption that feedback from others affects and shapes the moral self. Positive feedback on moral behavior increases an individual’s moral self-image, while negative feedback decreases an individual’s moral self-image [25]. The underlying cognitive process of the moral self involves mentalizing, which reflects the individual’s ability to understand and infer the mental states of others [26]. Numerous studies have identified the TPJ as a core brain region responsible for mentalizing [26,27] which is associated with moral self-evaluation.

Resting-state functional magnetic resonance imaging (RS-fMRI) captures the spontaneous neural activity of the brain (i.e., low-frequency fluctuations) without any specific task [28]. Here, we focused on two widely used indicators, the fractional amplitude of low-frequency fluctuations (fALFF) and the resting-state functional connectivity (RSFC). The fALFF reflects regional properties of intrinsic brain dynamics and has been found to be related to individual differences in cognition, emotion, and personality [29]. Meanwhile, the RSFC quantifies the synchrony of spontaneous neuronal signals across different regions [30]. This index has been shown to efficiently identify potential neural connections for risk propensity [31] and well-being [32].

The present study used fALFF and RSFC measures to investigate the shared and distinct neural bases of self-worth and moral ambivalence. To our knowledge, there have been no studies investigating which spontaneous activities and functional connectivity in brain regions during rest are related to self-worth and moral ambivalence. We propose a preliminary hypothesis that self-worth and moral ambivalence might be linked to the dACC, OFC, and CMS. The shaping of moral self-image is influenced by the perceptions of others, and moral ambivalence is also associated with the TPJ.

## 2. Methods

### 2.1. Participants

A total of 112 undergraduate students (42 males, age = 20.88 ± 1.48 years; 70 females, age = 21.11 ± 1.72 years) with no history of psychiatric disability and neurological impairment were recruited from SouthWest University via on-campus advertisements. The duration of education varied between 13 and 16 years. This study was approved by the local ethical committee of XX University (masked for review). All participants provided written informed consent before the study. They will receive corresponding compensation for their participation.

### 2.2. SAM Scale

The Self-Ambivalence Measure (SAM) [1] is a widely used measure in psychological and clinical research to assess conflicting self-perceptions. In a previous study, we revised the SAM in the Chinese population which consisted of 16 items and found that the three-factor structure of the SAM demonstrated a good fitting effect [9]. These items measure self-worth, moral ambivalence, and public self-consciousness. The reliability coefficients of the sub-dimensions of the SAM were acceptable (self-worth, McDonald’s ω = 0.91; moral ambivalence, McDonald’s ω = 0.92; and public self-consciousness, McDonald’s ω = 0.84) [33]. The revised SAM demonstrated convincing validity, evidenced by its strong positive correlation with dialectical thinking and Rosenberg self-esteem questionnaires. Participants indicated their degree of agreement with each statement on a 5-point scale (0 = not at all to 4 = agree totally). The higher the scores they reported, the greater the self-ambivalence.

### 2.3. RS-fMRI Data Acquisition

Functional and structural data were acquired on a 3T Prisma Siemens Trio MRI scanner at the Brain Imaging Centre of SouthWest University. The RS-fMRI scanning procedure used 242 gradient echo-planar imaging (EPI) volumes with the following parameters: repetition time (TR) = 2000 ms; echo time (TE) = 30 ms; slices = 62; slice thickness = 2 mm; resolution matrix = 64 × 64; flip angle = 90°; slice gap = 1 mm; field of view = 224 × 224 mm^2^; and voxel size = 2.0 × 2.0 × 2.0 mm^3^.

Every participant underwent an 8 min RS-fMRI scan, where they were instructed to stay motionless, keep their eyes closed, stay conscious, and not focus on anything particular. No participants reported falling asleep during the scan.

### 2.4. Data Preprocessing

The preprocessing was conducted using the Data Processing Assistant for Resting-State fMRI (DPARSF) toolbox [34] and SPM12. The initial 5 time points were removed to reduce the instability of the initial MRI readings. The remaining 237 volumes were adjusted for slice timing and realigned for head motion. Afterward, each subject’s fMRI images were registered to their segmented high-resolution T1-weighted anatomical images. The nuisance variables were regressed, and these included six head motion parameters, the white matter (WM) signal, and the cerebral spinal fluid (CSF) signal. fMRI images were normalized to the standard Montreal Neurological Institute (MNI) template using Diffeomorphic Anatomical Registration Through Exponentiated Lie algebra (DARTEL) [35]. The resolution voxel size of this normalization was 3 × 3 × 3 mm^3^, and a 4 mm full width at half maximum (FWHM) of Gaussian kernel was used for smoothing functional connectivity strength. This filter was set to a range of 0.01–0.1 Hz. However, it is important to note that this filtering process was applied only for RSFC and not for the fALFF. This method helps to enhance the quality of the images by reducing unwanted frequencies. Among all the participants, none exhibited head motion exceeding 2.5 mm in any direction.

#### 2.4.1. fALFF–Behavior Correlation Analyses

The time courses in each voxel, which represent the changes in signal intensity over time, were transferred into the frequency domain. The mean square root was then computed across a low-frequency range of 0.01–0.1 Hz. The fALFF was calculated as a fraction, which involved dividing the amplitudes within a low-frequency range (0.01–0.1 Hz) by the amplitudes across the entire frequency range (0.01–0.25 Hz). Last, the normalized score of the fALFF, known as z-fALFF, was obtained to exclude the global influences of variability between individuals. The computations for these processes were conducted using the DPARSF toolbox [34].

Whole-brain analyses were performed to investigate the relationship between the regional spontaneous functional activity and self-ambivalence. Multiple regression analyses were performed, taking into consideration the mean fALFF values and the subscale of self-ambivalence scores. In accordance with previous approaches, gender and age were controlled as covariates in the regression analyses. It used the Gaussian Random Field (GRF) program to correct for multiple comparisons. The threshold was set as a corrected cluster at *p* < 0.05 and a single voxel at *p* < 0.005, with a cluster size ≥ 25 voxels, and with the dimensions being 61 × 73 × 61, and two-tailed. These analyses were conducted using the DPABI toolbox (http://rfmri.org/DPABI).

#### 2.4.2. RSFC–Behavior Correlation Analyses

To investigate the interplay between brain regions observed in fALFF–behavior correlation analyses and other regions that correlate with self-worth and moral ambivalence, we performed seed-to-voxel functional connectivity analyses using the CONN connectivity toolbox [36]. Since the data have already been preprocessed, this step was omitted here. These regions of interest (ROI) were identified using clusters that showed a significant relationship to self-ambivalence. The time series of all voxels in each ROI were averaged for each subject and related to the time series of other voxels in the brain. We conducted correlation analyses between the correlation maps and self-worth and moral ambivalence scores to detect the relationship between strength connectivities and self-ambivalence, with age and gender serving as controlling variables. The results were corrected using a cluster-wise threshold of *p* < 0.05 (False Discovery Rate, FDR-corrected) and a voxel-wise threshold of *p* < 0.001. Furthermore, we also conducted ROI-ROI functional analyses to examine whether CMS is associated with cognitive processes of self-ambivalence. These ROIs were derived from 246 templates including the bilateral MPFC, PCC, ACC (ventral part), and precuneus, respectively. The statistical tests were automatically corrected for multiple comparisons using FDR (*p* < 0.05).

## 3. Results

### 3.1. Behavior Data

The mean scores for self-worth and moral ambivalence were 9.94 (standard deviation, SD = 5.41) and 6.34 (SD = 5.47). A Pearson correlation analysis showed that self-worth ambivalence was positively associated with moral ambivalence (*r* = 0.67, *p* < 0.001), which suggests that participants with higher self-worth ambivalence were associated with higher moral ambivalence. Next, we will investigate the spontaneous brain activity of self-worth ambivalence and moral ambivalence.

### 3.2. Brain Regions and Functional Connectivity Related to Self-Worth Ambivalence

**Figure 1:**
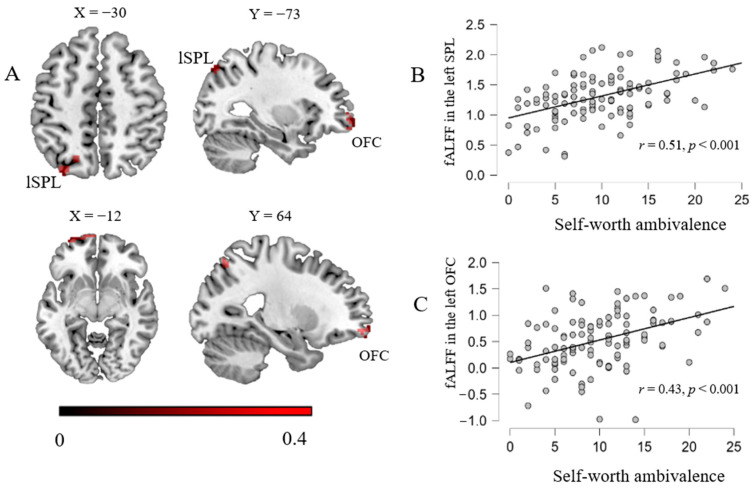
Brain regions linked with self-worth ambivalence (**A**). Color bars represent R-values. (**B**,**C**) show positive scatter plots between self-worth ambivalence scores and fALFF in left superior parietal lobule and left OFC, respectively. Note: OFC = orbitofrontal cortex; lSPL = left superior parietal lobule.

To explore the relationship between fALFF and self-worth ambivalence, we performed a regression analysis. The results found that self-worth ambivalence was positively correlated with zfALFF in the OFC and superior parietal lobule and negatively correlated with zfALFF in the posterior lobe of the cerebellum, occipital lobe, and parietal lobe (Table 1; Figure 1).

**Figure 2:**
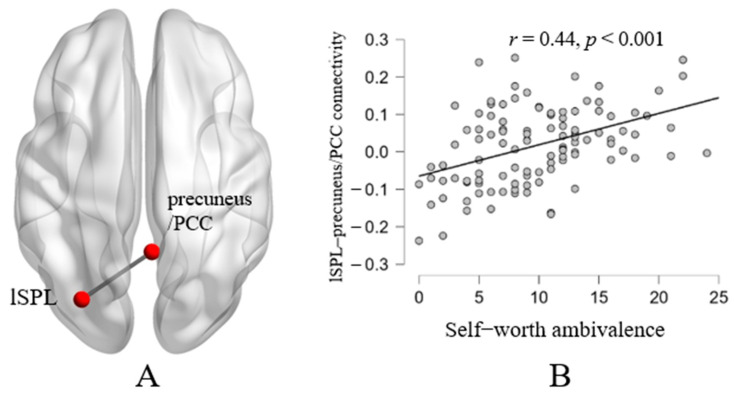
Functional connectivity linked with self-worth ambivalence. Self-worth ambivalence was positively related to the RSFC between the left superior parietal lobule (seed) and precuneus/posterior cingulate cortex (**A**). Scatter plots depicting a correlation between self-worth ambivalence and the connectivity between the left SPL and precuneus/PCC (**B**).

Moreover, to reveal whether the identified brain regions interact with other regions that could be associated with self-worth ambivalence, functional connectivity strength behavior correlation analyses were performed. Age and gender were taken into account as nuisance variables. The brain regions that exhibited significant positive correlations with self-worth ambivalence were utilized as seed regions in the RSFC analysis, including the left superior parietal lobule and orbitofrontal cortex. The results indicated that self-worth ambivalence was positively related to the RSFC strength between the SPL and precuneus/PCC (Table 2; Figure 2). In other words, the higher the scores of self-worth ambivalence, the greater the connection strength between the left SPL and precuneus/PCC. With the orbitofrontal cortex as the seed region, no significant results were identified.

Functional connectivity analyses of ROI-to-ROI showed positive relationships for functional connectivity between the OFC and bilateral MPFC, and the left PCC and left precuneus with self-worth ambivalence. The higher the self-worth ambivalence scores, the greater the strengths of functional connectivity between the OFC and these regions. It is worth noting that these functional connections are marginally significant after FDR correction (*p*_uncorrected_ < 0.05, *p*-FDR = 0.07).

### 3.3. Brain Regions and Functional Connectivity Related to Moral Ambivalence

**Figure 3:**
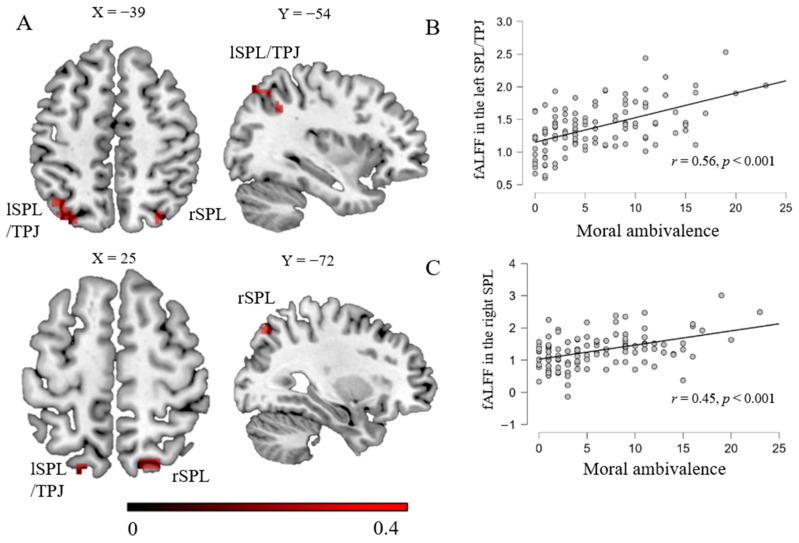
Brain regions linked with moral ambivalence (**A**). Color bars represent R-values. (**B**,**C**) show positive scatter plots between moral ambivalence scores and fALFF in bilateral superior parietal lobule, respectively. Note: lSPL = left superior parietal lobule; rSPL = right superior parietal lobule; TPJ = temporoparietal junction.

**Figure 4:**
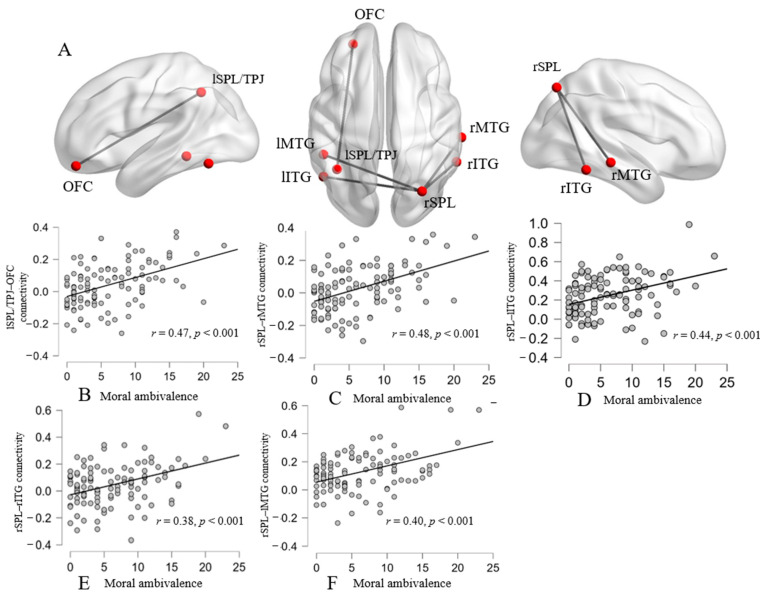
Functional connectivity linked with moral ambivalence. Moral ambivalence was positively associated with the strength of the RSFC between the left superior parietal lobule/ temporal-parietal junction (seed) and orbitofrontal cortex and between the right superior parietal lobule (seed) and bilateral middle temporal gyrus and bilateral inferior temporal gyrus (**A**). Scatter plots depicting correlations between moral ambivalence and the strength of lSPL-OFC, rSPL/TPJ-rMTG, rSPL/TPJ-lITG, rSPL/TPJ-rITG, and rSPL/TPJ-lMTG respectively (**B**–**F**). Note: OFC = orbitofrontal cortex; SPL = superior parietal lobule; MTG = right middle temporal gyrus; ITG = inferior temporal gyrus; TPJ = temporal–parietal junction.

Next, a similar regression analysis was used to explore the relationship between the fALFF and moral ambivalence. The results indicated that moral ambivalence was positively related to the zfALFF in the bilateral superior parietal lobule. It is noteworthy that a region of the left SPL (superior parietal lobule) extends to the left temporoparietal junction (TPJ) (Table 3; Figure 3). These regions served as seed regions for subsequent RSFC analysis. The results revealed that moral ambivalence was positively associated with the RSFC strength between the left SPL/TPJ and OFC, postcentral gyrus, and cerebellum (Table 4; Figure 4). That is, the higher the score of moral ambivalence, the stronger the RSFC strengths between the seed and these brain regions. When the right SPL served as a seed, the RSFC strengths between the SPL and specific regions were positively associated with moral ambivalence. These regions include the bilateral middle temporal gyrus and bilateral inferior temporal gyrus.

The ROI-to-ROI functional connectivity analyses were used to examine whether CMS was associated with the potential cognitive processing of moral ambivalence. The results showed positive correlations between functional connectivity between the right SPL/TPJ and the right precuneus and right PCC with moral ambivalence. The higher the moral ambivalence scores, the greater the strengths of functional connectivity between the SPL/TPJ and these brain regions. However, these functional connections have not been FDR-corrected (*p*_uncorrected_ = 0.06, *p*-FDR = 0.29; *p*_uncorrected_ = 0.08, *p*-FDR = 0.29). There were no obvious correlations between moral ambivalence and the strengths of functional connectivity between the left SPL and these brain regions.

## 4. Discussion

The present study conducted the fALFF–behavior correlation analyses and seed-based RSFC analyses to reveal the neural bases of self-worth and moral ambivalence. Consistent with previous research, a significant positive relationship between self-worth and moral ambivalence was observed [2]. The whole-brain correlation analyses indicated that higher self-worth ambivalence was related to a higher fALFF in the left SPL and left OFC and a lower fALFF in the right parietal lobe, left occipital lobe, and right cerebellum posterior lobe. Further RSFC analyses revealed that higher self-worth ambivalence was related to greater connectivity strength between the left SPL as a seed and the precuneus/PCC. Subsequent ROI-to-ROI functional connectivity analyses revealed that the strengths of functional connectivity between the OFC and CMS (including bilateral MPFC, left PCC, and left precuneus) were marginally significantly correlated with self-worth ambivalence. Meanwhile, the scores of moral ambivalence were correlated with the fALFF in the left SPL (extended into TPJ) and right SPL. The results of the RSFC indicated that higher moral ambivalence was associated with greater functional connectivity between the left SPL/TPJ as a seed and the OFC, as well as greater functional connectivity between the right SPL as a seed and the bilateral middle and inferior temporal gyrus.

Correlation analysis results found that fALFFs in the SPL were positively related to self-worth and moral ambivalence, suggesting that the SPL is a core brain region for ambivalence. The SPL is an integral part of the dorsal attention network and is activated in various cognitive tasks [37,38]. It supports top-down attentional processing and is associated with arousal and vigilance [39]. Indeed, behavioral studies have found that ambivalence can help individuals overcome cognitive traps [40,41], such as the framing effect and attribution biases [42], thereby leading to more effective decision-making. Some studies even found that ambivalence would increase judgment accuracy and enhance creativity [43,44]. The potential reason for this could be that ambivalence provides individuals with signals of contradiction, serving to alert them to the complex and conflicting elements in their environment [45]. Hence, it is understandable that individuals with higher ambivalence exhibit increased alertness, thus prompting the involvement of the SPL.

The results also found that the fALFF in the OFC was positively related to self-worth ambivalence, and functional connectivity between the OFC and left SPL/TPJ was positively related to moral ambivalence. This indicated that the OFC is an important region related to ambivalence [17]. It is worth noting that the function of the OFC in the previous study was to resolve conflicts. However, the OFC was also activated when it was not necessary to make a dichotomous judgment of an ambivalent stimulus [18]. This implies that the OFC might not necessarily be used to resolve conflicts. Numerous studies have demonstrated that the OFC involves the computation of the subjective value of stimuli of abstract rewards and punishments, such as praise or losing money [46,47,48]. The premise of ambivalence is that individuals are exposed to both positive and negative information at the same time. Research has confirmed that the OFC was recruited when people receive positive and negative feedback from others [49,50]. It is likely that the OFC is responsible for representing the subjective value of different valence information in ambivalence processing. This requires further support from more empirical research in the future. Notably, the results did not report a significant region in the dACC. Due to the limited number of participants, the statistical power was inadequate to identify significant activity in the dACC. When correction thresholds in the fALFF correlation analyses were relaxed, activity in the dACC could be observed.

Functional connectivity analyses revealed that the CMS was associated with the neural bases of self-worth and moral ambivalence. The CMS has been suggested to be the underlying neural basis of self-evaluation, such as the involvement in self-referential processing [51]. The precuneus/PCC are important regions in the CMS, and their activity is responsible for the extraction of personal episodic memory [52]. Previous studies have found that the strength of functional coupling between the dACC and PCC in the resting state is related to the dialectical self, which reflects an individual’s acceptance of conflict and inconsistency [53]. Other studies have revealed that the PCC/precuneus are related to moral cognition, such as engaging in the processing of moral decisions [54]. However, the specific function of the PCC in the cognitive process of moral ambivalence could not be determined in the present study, and future research could further answer this question through an experimental design. The results were not significant after multiple comparison corrections (FDR), which requires future research to increase the power by increasing the sample size.

Consistent with our assumption, it was found that the TPJ engaged in the specific brain areas for moral ambivalence. The TPJ, as a key region of mentalizing, plays an important role in understanding others’ thoughts, intentions, and beliefs [26,55]. It was found that the TPJ engaged in moral self-evaluation. In addition, the middle and inferior temporal gyrus are also engaged in the processing of mentalizing, which is responsible for processing mental inferences about others [27]. Many studies underscore the role of interpersonal interactions in the formation of a moral self-image [56]. For example, Jordan found that an individual’s moral self-image is influenced by feedback received from others [25]. Therefore, the perspectives of others are an important avenue for shaping one’s moral self-image.

Several limitations in the current study should be tackled in upcoming research. First, although we obtained self-ambivalence-related brain regions through regression analysis, which deepened the understanding of ambivalence, the specificity of the results has yet to be tested. Because self-ambivalence is a complex concept, it has often been confused in previous studies with, for example, self-esteem and the dialectical self, which can also bring about negative emotions. Future studies could identify self-ambivalence-specific brain regions through more rigorous manipulation. Second, the present research adopted the fALFF and RSFC to measure the brain’s function. It is necessary for future research to consider other measures (e.g., regional gray matter volume) to investigate the neural basis of self-ambivalence. This could ensure a more comprehensive understanding and knowledge of self-ambivalence. Third, ambivalence is closely related to culture, and individuals from different cultures have varying degrees of tolerance for ambivalence [57,58]. Culture shapes the function of the human brain [59], thus future research needs to examine the neural basis of self-ambivalence under different cultures to enhance our understanding of it. Fourth, the sample population of this study primarily consists of healthy college students. Caution should be exercised when generalizing the conclusions to other populations, especially individuals with obsessive–compulsive disorder and schizophrenia. This is due to research indicating abnormal neural activity in these clinical populations when processing ambivalent stimuli [60].

## 5. Conclusions

The current study explored the neural bases of self-worth and moral ambivalence, finding associations with spontaneous activity in the SPL and OFC. This suggests that attentional alertness and value representation underlie the cognitive processes of both. Subsequent functional connectivity analyses identified the strengths of connections between ROIs and the CMS, particularly the precuneus/PCC, which is related to both self-worth and moral ambivalence, although these results did not survive multiple comparison corrections. These findings suggest to some extent that self-evaluation is an underlying cognitive process of both. Additionally, morality was found to be associated with spontaneous activity in the TPJ, revealing mentalizing as a unique cognitive process for moral ambivalence. Together, our study provides the first evidence for the neural basis of self-worth and moral ambivalence and offers insights into the underlying cognitive processes of both constructs.

## Figures and Tables

**Table 1:** Regions where fALFFs were significantly linked with self-worth ambivalence.

Brain Regions	Peak	MNI	Coordinates	Peak *r*-Value	No. of Voxels
	x	y	z		
Left OFC	−12	69	−3	0.37	56
Left superior parietal lobule	−30	−75	48	0.35	34
Right cerebellum posterior lobe	30	−90	−27	−0.40	31
Left occipital lobe	−3	−93	27	−0.38	32
Right parietal lobe	6	−75	54	−0.40	58

Note: OFC = orbitofrontal cortex.

**Table 2:** Regions where RSFCs were significantly associated with self-worth ambivalence.

Seed	Brain Regions	Peak	MNI	Coordinates	Cluster *p* FDR	Peak	No. of Voxels
		x	y	z		p unc	
**lSPL**							
	Right precuneus/PCC	4	−52	36	<.01	<0.001	85
**OFC**							
	/						

Note: p unc = p uncorrected; OFC = orbitofrontal cortex; lSPL = left superior parietal lobule; PCC = posterior cingulate cortex.

**Table 3:** Regions in which zfALFFs were significantly linked with moral ambivalence.

Brain Regions	Peak	MNI	Coordinates	Peak *r*-Value	No. of Voxels
	x	y	z		
Left superior parietal lobule/TPJ	−39	−54	42	0.42	49
Right superior parietal lobule	30	−72	51	0.37	29

Note: TPJ = temporoparietal junction.

**Table 4:** Regions where RSFCs were significantly linked with moral ambivalence.

Seed	Brain Regions	Peak	MNI	Coordinates	Cluster *p* FDR	Peak	No. of Voxels
		x	y	z		*p* Unc	
**lSPL/TPJ**							
	Left OFC	−26	48	−18	<0.01	<0.001	120
	Left postcentral gyrus	−56	−6	32	<0.05	<0.001	65
	Left cerebellum	−32	−52	−58	<0.05	<0.001	66
**rSPL**							
	Right middle temporal gyrus	70	−28	−10	<0.001	<0.001	162
	Left inferior temporal gyrus	−56	−60	−28	<0.05	<0.001	74
	Right inferior temporal gyrus	60	−48	−16	<0.05	<0.001	73
	Left middle temporal gyrus	−56	−42	−12	<0.05	<0.001	71

Note: OFC = orbitofrontal cortex; lSPL = left superior parietal lobule; rSPL = right superior parietal lobule; TPJ = temporoparietal junction.

## Data Availability

The data used in the present study are available upon direct request by contacting the corresponding author due to the expensive cost of collecting research data and the involvement of participants’ personal information.
